# Effect of a 3-month L-carnitine supplementation and resistance training program on circulating markers and bone mineral density in postmenopausal women: a randomized controlled trial

**DOI:** 10.1186/s12986-023-00752-1

**Published:** 2023-08-02

**Authors:** Robert A. Olek, Emilia Samborowska, Piotr Wisniewski, Pawel Wojtkiewicz, Krystian Wochna, Jacek Zielinski

**Affiliations:** 1 Department of Athletics, Strength, and Conditioning, Poznan University of Physical Education, Krolowej Jadwigi 27/39, Poznan, 61-871 Poland; 2 Mass Spectrometry Laboratory, Institute of Biochemistry and Biophysics, Polish Academy of Sciences, Warsaw, Poland; 3 Chair and Department of Endocrinology and Internal Medicine, Medical University of Gdansk, Gdansk, Poland; 4 Endoscopy and Metabolic Disorders, Seventh Navy Hospital, Gdansk, Poland; 5 Department of Swimming and Water Lifesaving, Poznan University of Physical Education, Poznan, Poland

**Keywords:** Osteonectin, SPARC, Decorin, Trimethylamine N-oxide, Osteopontin, Sclerostin

## Abstract

**Background:**

Higher circulating levels of trimethylamine N-oxide (TMAO), which is a metabolite that can be produced by the gut microbiota from L-carnitine (LC), have been associated with bone mineral density (BMD). Because LC supplementation can improve bone density and microstructural properties in animal models, this study aimed to examine the effects of 12 weeks of LC supplementation on BMD and selected blood markers involved in bone metabolism of postmenopausal women participating in a resistance training (RT) program.

**Methods:**

Twenty-seven postmenopausal women, who had not been treated for osteoporosis, with a total T-score above − 3.0 and no diet differences completed 12 weeks of RT. The participants’ diets were supplemented with either 1 g of LC-L-tartrate and 3 g of leucine per day (LC group) or 4 g of leucine per day as a placebo (PLA group), in a double-blind fashion.

**Results:**

After the intervention in the LC group, plasma total carnitine and serum decorin levels were higher than the corresponding preintervention values (p = 0.040 and p = 0.042, respectively). Moreover, plasma TMAO and serum SPARC levels were higher in the LC group than the corresponding postintervention values in the PLA group (p < 0.001 and p = 0.030, respectively). No changes in the BMD were observed after 3 months of the intervention.

**Conclusions:**

Twelve weeks of LC supplementation during RT program increased plasma TMAO levels and appeared to affect signaling molecules, as indicated by the increase in the resting SPARC and decorin levels, with no significant modification in the BMD.

**Trial registration:**

Retrospectively registered at the ClinicalTrials.gov (NCT05120011).

**Supplementary Information:**

The online version contains supplementary material available at 10.1186/s12986-023-00752-1.

## Introduction

Osteoporosis is a complex, multifactorial condition characterized by the loss of bone mineral density (BMD) leading to an increased susceptibility to fractures. The prevalence of osteoporosis increases with age and is higher among older women [1]. According to numerous studies, resistance training (RT) intervention may significantly preserve bone mass or avert bone loss in women [2]. These effects are not only dependent on mechanical load, because the skeletal muscle may serve as an endocrine organ that is capable of secreting cytokines to modulate bone metabolism [3, 4]. Even 3 months of an exercise training program can increase circulating markers of bone formation in postmenopausal women [5].

Changes in bone metabolism, BMD, a high risk of recurrent falls, or a combination of these factors are potentially related to nutritional factors [6]. Several studies, using an aging ovariectomized rat model of osteoporosis, have shown that L-carnitine (LC) supplementation can improve bone density and microstructural properties [7–9]. These findings indicated a reduction in bone resorption rate and a decrease in mineral turnover by LC [7–9]. In addition, an improvement in the levels of inflammatory biomarkers was demonstrated. The LC treatment led to a decrease in the serum levels of tumor necrosis factor-α (TNF-α) and interleukin-6 (IL-6) [8, 9].

Moreover, recent studies have shown a strong relationship between bone metabolism and intestinal microbiota, and their potential effect on the risk of developing osteoporosis [10]. Furthermore, it is well known that LC is metabolized by the gut microbes to trimethylamine (TMA), which is converted to TMA N-oxide (TMAO). Thus, LC treatment increases plasma TMAO levels [11]. A tenfold increase in fasting plasma TMAO levels was noted following 3 months of oral LC supplementation in healthy older women [12]. Interestingly, TMAO affects bone homeostasis [13]. The decline in plasma TMAO levels was related to a greater reduction in BMD during a weight loss program in overweight and obese participants, suggesting that TMAO might protect against decreases in BMD [14]. In contrast, elevated TMAO levels were associated with hip fractures [15, 16].

Therefore, this study aimed to establish whether the increase in plasma TMAO levels induced by LC supplementation affects the BMD and selected blood markers of older women involved in an RT program. We hypothesized that changes in plasma TMAO levels related to LC supplementation affect bone metabolism.

## Methods

### Ethics approval and consent to participate

This study was conducted in accordance with the Declaration of Helsinki. The study protocol was approved by the Independent Bioethics Committee for Scientific Research at the Medical University of Gdansk (NKBBN/354 − 201/2017) and registered in the ClinicalTrials.gov Registry (NCT05120011). Before starting the experimental procedure, all participants were informed about the procedure, risks, and expected outcomes and they provided their written informed consent for participation.

### Sample size

Previously reported results [12] were adopted in two-way ANOVA sample size calculation using Statistica 13.1 software (Dell Inc., Tulsa, OK, USA). Means and standard deviations of plasma TMAO in the supplemented and placebo groups, before and after three months of LC treatment, were applied with the alpha level and the power of the test set at 0.05 and 0.95, respectively. On the basis of these parameters, the required sample size has been obtained equal to 9.

### Participants

**Fig. 1:**
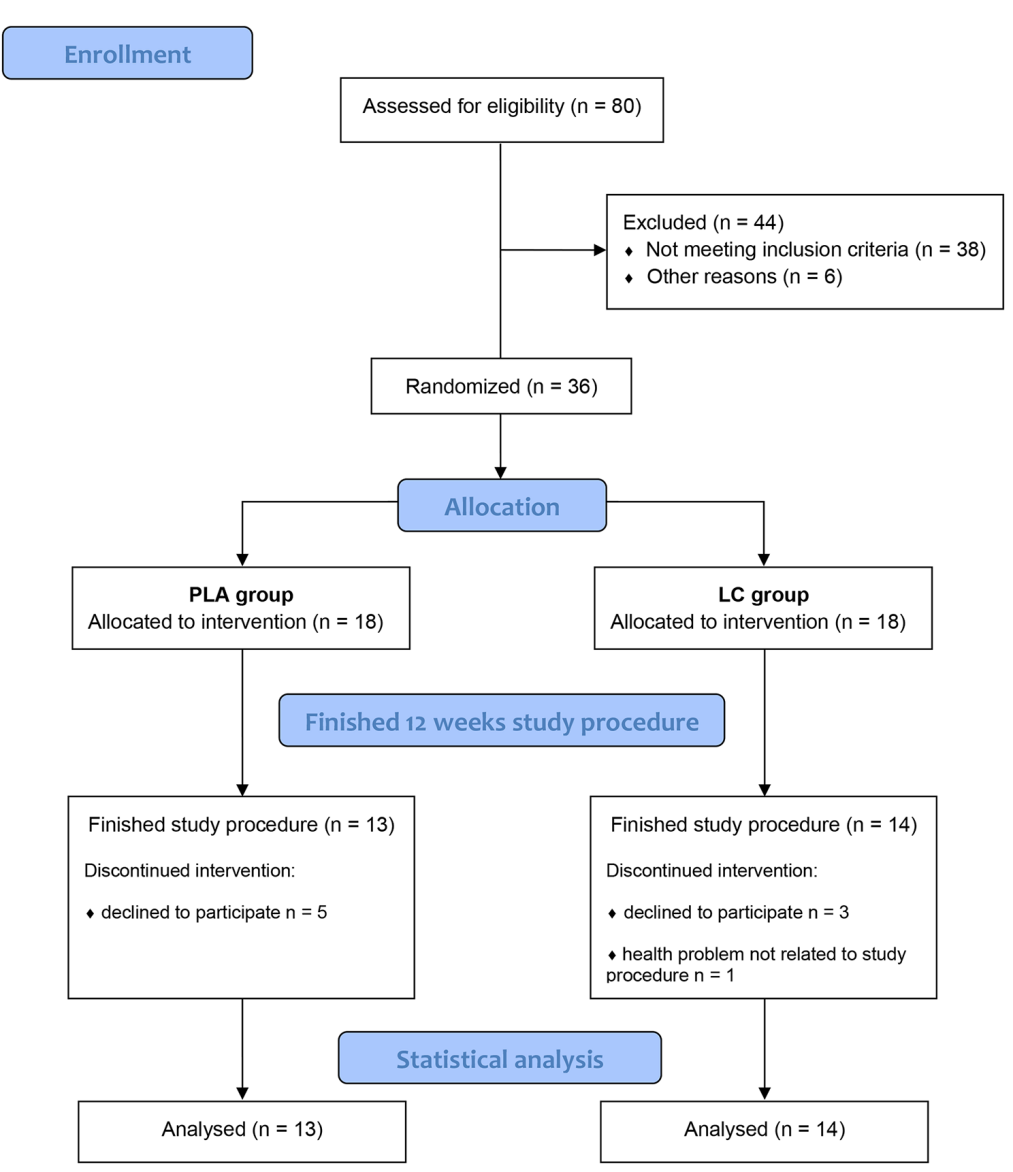
Flow chart of participant recruitment and participation in the study

Postmenopausal women, with no cardiovascular disease, liver or kidney disease, gastrointestinal disorder, including stomach ulcer or erosions, cancer, diabetes, musculoskeletal disease, and other severe chronic diseases were recruited through local advertisements. All included participants presented a physician’s certificate indicating a lack of contradictions to strength training. Thirty-six women without a history of osteoporosis, low-energy fractures, or antiresorptive treatment were included in the study (Fig. 1).

### Experimental design and study procedure

Participants were randomly assigned (1:1 ratio) to one of the two groups using the Random Sequence Generator (RANDOM.ORG, Dublin, Ireland). The randomization sequence was stored by a researcher who had no contact with the participants. Over the 12 weeks, the participants were supplemented with either 1 g of LC-L-tartrate and 3 g of leucine per day (LC group) or 4 g of leucine per day as a placebo (PLA group), in a double-blind fashion. The supplements were encapsulated in identical gelatin capsules, and the participants were instructed to consume the supplements once a day with their main meal.

### RT protocol

During the study period, all subjects participated in the RT program, which was held in a commercial gym, twice a week. Each participant attended the program on Mondays and Wednesdays, or Tuesdays and Thursdays. Professional coaches conducted each training session. Over the 12 weeks, 24 training sessions, each lasting for 45–60 min were performed for each group. The RT protocol was based on a previously described procedure [17]. Each training session started with a 10-min warm-up on a treadmill (walking). The participants then performed three sets of the following four exercises: leg press, leg extension, shoulder press or horizontal row, and chest press or lateral pulldown. The leg press and extension were performed at every training session, but the shoulder press and lateral pulldown were performed only on Monday or Tuesday, and the horizontal row and chest press only on Wednesday or Thursday. Each session ended with a 10-min cooldown on a cycle ergometer.

Before starting the training protocol, a one-repetition maximum (RM) test was performed. During the first 2 weeks, the workload was set at 65% of RM for each exercise, and the exercise was performed in three sets of 10–12 repetitions. After 2 weeks, the workload was increased to 80% of RM, and each exercise was performed in three sets of 6–8 repetitions.

### Blood collection and analysis

During the week before initiation of the experimental protocol and 12 weeks after the initiation, fasting blood samples were taken from the antecubital vein into BD Vacutainer tubes (Becton, Dickinson and Company, Franklin Lakes, NJ, USA). After collection, the samples were centrifuged at 2000 g and 4 °C for 10 min, and aliquots were stored at -80 °C for later analyses. Plasma TMAO, and total carnitine (TC) levels were measured by the liquid chromatography-mass spectrometry system at the Mass Spectrometry Laboratory, Institute of Biochemistry and Biophysics, Polish Academy of Sciences (Warsaw, Poland) as described previously [18, 19]. Serum dickkopf-1 (DKK1), osteoprotegerin (OPG), osteopontin (OPN), sclerostin (SOST), and fibroblast growth factor-23 (FGF-23) levels were determined using the MILLIPLEX Human Bone Magnetic Bead Panel (catalog number [cat. #]: HBNMAG-51 K; Merck KGaA, Darmstadt, Germany). Insulin-like growth factor-1 (IGF-1), IL-6, TNF-α, decorin, and osteonectin/secreted protein acidic and rich in cysteine (SPARC) levels were measured using commercially available enzyme immunoassay kits (total IGF-1, cat. # DG100; IL-6, cat. # HS600B; TNF-α, cat. # HSTA00E; decorin, cat. # DY143 and # DY008; SPARC, cat. #DY941-05; R&D Systems, Minneapolis, MN, USA), and C-reactive protein (CRP) level using cat. # EIA-3954 kit (DRG Instruments GmbH, Marburg, Germany).

### Dual-energy X-ray absorptiometry (DXA)

Areal BMDs of the lumbar spine (L1–L4), hip, and whole body were measured on separate days, at baseline and after 12-week RT. Measurements were performed using a Hologic Discovery Wi DXA scanner and analyzed using APEX software version 13.4. All DXA scans were performed by the same trained technician according to the manufacturer’s instructions, and analyzed by the same investigator who was blinded to the intervention. T-scores and Z-scores were calculated using the manufacturer’s reference ranges.

### Diet

Three-day food records were self-reported for 2 weekdays and 1 weekend day at the beginning of the study. Participants were instructed to note the amounts of food and beverages consumed. The diet was analyzed in terms of the amount of energy, protein, carbohydrates, and fat consumed.

### Statistical analysis

Participants included in the statistical analyses completed a minimum of 80% of the training sessions. All calculations were performed using Statistica 13.1 software (Dell Inc., Tulsa, OK, USA). Baseline characteristics and dietary composition of the participants were compared using Student’s t-test. To examine the treatment and time interaction in blood markers, BMD data, and strength variables two-way analysis of variance (ANOVA) for repeated measurements was performed. In case ANOVA yielded a significant effect, Tukey’s HSD test was used for post hoc comparisons. Correlations between the changes in absolute values from before to after the RT intervention were calculated using Pearson and Spearman correlation tests for normally and nonnormally distributed data, respectively. A probability level of p < 0.05 was considered significant. All data are expressed as mean ± standard deviation, unless otherwise stated.

## Results

The study protocol was completed by 27 participants (Fig. 1); their characteristics are presented in Table 1.

**Table 1:** Baseline characteristics of participants. Data are presented as means ± SD

	PLA (n = 13)	LC (n = 14)	p-value
Age (years)	67.9 ± 2.3	68.1 ± 2.3	0.808
Age of menopause (years)	53.6 ± 3.7	52.5 ± 3.0	0.399
Height (cm)	159.8 ± 4.1	159.7 ± 6.7	0.980
Body mass (kg)	72.9 ± 13.0	74.3 ± 14.9	0.788
Total body T-score	-1.323 ± 1.232	-1.093 ± 0.793	0.566
Total body Z-score	-0.115 ± 0.886	0.064 ± 0.537	0.526
Lumbar spine T-score	-1.000 ± 1.105	-0.508 ± 0.881	0.237
Lumbar spine Z-score	1.009 ± 1.155	1.423 ± 0.820	0.317
Total hip T-score	-0.423 ± 1.069	-0.250 ± 0.638	0.611
Total hip Z-score	0.992 ± 1.029	1.171 ± 0.632	0.587
Hip neck T-score	-0.977 ± 0.858	-0.936 ± 0.685	0.891
Hip neck Z-score	0.715 ± 0.852	0.786 ± 0.664	0.812

**Fig. 2:**
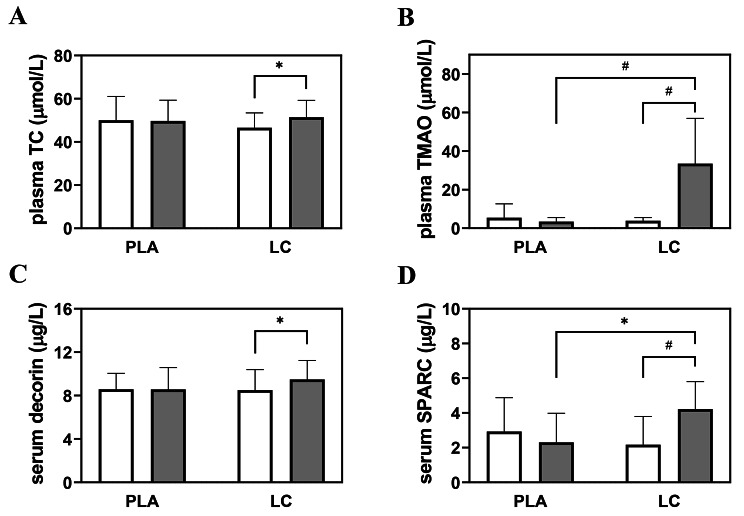
Plasma total carnitine (panel A), TMAO (panel B), and serum decorin (panel C), SPARC (panel D) concentrations in the PLA and LC groups before (white bars) and after 12 weeks of intervention (gray bars). Values are presented as means (± SD); # p < 0.001; * p < 0.05

After the intervention in the LC group, plasma TC (Fig. 2A) and serum decorin (Fig. 2C) levels were higher than the corresponding preintervention values (p = 0.040 and p = 0.042, respectively). Moreover, plasma TMAO (Fig. 2B) and serum SPARC (Fig. 2D) levels were higher in the LC group than the corresponding postintervention values in the PLA group (p < 0.001 and p = 0.030, respectively). No differences in circulating OPN, OPG, SOST, FGF-23, DKK1, IGF-1, IL-6, TNF-α, and CRP levels were noted (Table 2). Lumbar spine BMD tended to decrease in the LC group after the 3-month intervention (p = 0.069), but no other changes in BMD were noted (Table 3). Significant improvement in RM in time was observed with the seated leg press (p < 0.001), leg extension (p < 0.001), seated chest press (p < 0.001), shoulder press (p < 0.001), lateral pulldown (p < 0.001), cable row (p < 0.001), and total (p < 0.001), with no differences between the groups (Table 4).

**Table 2:** Serum biomarkers in the PLA and LC groups before and after 12 weeks of intervention. Data are presented as means ± SD

	PLA		LC		Group x Time (p)
	pre	post	pre	post	
OPN (µg/L)	19.8 ± 3.8	18.6 ± 2.8	18.4 ± 5.2	17.3 ± 6.2	0.954
SOST (ng/L)	710 ± 445	704 ± 470	764 ± 500	647 ± 362	0.367
FGF-23 (ng/L)	22.6 ± 21.3	25.8 ± 23.3	21.9 ± 18.0	22.9 ± 15.7	0.400
OPG (ng/L)	142 ± 54	149 ± 51	146 ± 57	141 ± 44	0.460
DKK1 (ng/L)	69.9 ± 14.3	69.9 ± 28.1	75.4 ± 26.5	74.4 ± 26.4	0.910
IGF-1 (µg/L)	77.7 ± 14.5	82.9 ± 20.5	79.5 ± 25.9	85.3 ± 26.7	0.941
IL-6 (ng/L)	1.6 ± 0.6	1.9 ± 0.8	1.8 ± 1.1	1.9 ± 1.4	0.566
TNF-α (ng/L)	1.2 ± 0.3	1.1 ± 0.2	1.0 ± 0.3	1.0 ± 0.3	0.478
CRP (mg/L)	2.3 ± 1.7	2.3 ± 2.1	2.3 ± 2.3	2.3 ± 2.4	0.970

**Table 3:** BMD values in the PLA and LC groups before and after 12 weeks of intervention. Data are presented as means ± SD

	PLA		LC		Group x Time (p)
	pre	post	pre	post	
Lumbar spine (L1-L4)	0.975 ± 0.150	0.978 ± 0.143	0.991 ± 0.113	0.970 ± 0.102 ^#^	0.048
Total hip	0.889 ± 0.132	0.888 ± 0.134	0.913 ± 0.077	0.912 ± 0.081	0.945
Femoral neck	0.740 ± 0.095	0.744 ± 0.093	0.749 ± 0.074	0.737 ± 0.071	0.071
Subtotal body	0.940 ± 0.075	0.947 ± 0.076	0.958 ± 0.064	0.952 ± 0.063	0.221
Total body	1.004 ± 0.092	1.016 ± 0.095	1.021 ± 0.061	1.021 ± 0.063	0.280

# p < 0.1 comparing to pre in the same group

**Table 4:** RM in the PLA and LC groups before and after 12 weeks of intervention. Data are presented as means ± SD

	PLA		LC		Group x Time (p)
	pre	post	pre	post	
Seated Leg Press (kg)	72.8 ± 26.7	81.6 ± 23.9	70.5 ± 13.5	88.9 ± 12.2	0.052
Leg extension (kg)	45.4 ± 11.0	51.0 ± 13.2	46.2 ± 8.0	53.0 ± 10.5	0.623
Seated chest press (kg)	18.9 ± 10.7	22.5 ± 9.6	19.5 ± 6.5	23.9 ± 5.2	0.568
Shoulder press (kg)	17.4 ± 10.5	21.2 ± 8.6	23.1 ± 8.2	25.5 ± 7.7	0.215
Lateral pull-down (kg)	24.7 ± 5.1	30.9 ± 3.3	25.0 ± 5.0	30.4 ± 5.1	0.649
Cable row (kg)	32.3 ± 7.8	38.8 ± 7.4	31.1 ± 5.9	34.6 ± 6.3	0.196
Total (kg)	212 ± 62	246 ± 55	215 ± 25	256 ± 21	0.320

We observed a positive correlation between the changes in serum decorin and SPARC levels (r = 0.543, p = 0.003; Fig. S1A), but only changes in the decorin level correlated with total BMD (r = 0.383, p = 0.049; Fig. S1B). Changes in the levels of both circulating biomarkers were correlated with the TMAO levels (rho = 0.568, p = 0.002; Fig. S2A and rho = 0.574, p = 0.002; Fig. S2B, for SPARC and decorin, respectively).

No differences were noted in the participants’ diets (Table S1).

## Discussion

Plasma TMAO reached a level that was comparable with the previously reported level following 12 weeks of supplementation in older women [12], despite the administration of a lower LC dose. However, changes in the TMAO level were not associated with BMD. The combination of LC supplementation and RT increased circulating SPARC and decorin levels but did not affect the other studied markers. Interestingly, changes in SPARC and decorin levels correlated with the TMAO level.

Physical activity may alter the secretion of signaling proteins from skeletal muscles [20]. For example, the SPARC level increased in the skeletal muscles of young male subjects after 11 weeks of a strength-training program [21], and it transiently increased in the serum of young healthy men immediately after a single bout of cycling [22]. In addition, 4 weeks of training at 70% of maximal oxygen uptake significantly promoted the exercise-induced increase in serum SPARC level. In contrast, resting serum SPARC level was not modified between pre- and post-training [22]. Our findings suggest that LC supplementation may have augmented SPARC secretion in the postmenopausal women who participated in the RT program, because such an effect was not observed in the PLA group participants who completed the same RT protocol.

SPARC plays a critical role in maintaining bone mass and quality [23]. Plasma SPARC levels positively correlate with lumbar spine BMD, and 12 months of recombinant human parathyroid hormone (1–34) treatment in osteoporotic patients increases the circulating SPARC level, which is associated with changes in lumbar BMD at L2-L4 [24]. The mechanisms by which SPARC affects bone formation, maintenance, and repair might occur through multiple pathways, including the regulation of procollagen processing and assembly in the bone matrix, cross-linking, mineralization, and/or osteoblast/osteoclast differentiation and activity [25]. Changes in SPARC level in our study were correlated with changes in circulating levels of decorin, which is a collagen-associated extracellular matrix proteoglycan that promotes the formation of bone matrix and calcium deposition to regulate bone morphogenesis [26].

The positive effect of LC supplementation on bone mineral turnover has been indicated in animal studies [7–9]. Moreover, LC administration may prevent the loss of BMD in patients with chronic kidney disease [27], chronic liver disease [28], and pemphigus vulgaris [29]. The anti-inflammatory effect of LC has been suggested as a potential mechanism underlying these findings [8, 9, 28]. However, we observed no changes in the levels of inflammatory biomarkers, which is similar to the findings for a previously reported 6-month LC treatment in healthy older women [30]. Furthermore, levels of other well-known bone turnover markers such as DKK-1, SOST, OPG, and OPN [31–34] were not changed in the present study. In addition, higher circulating SPARC and decorin levels were not associated with changes in BMD. Despite the recently reported positive [14] and negative [15, 16] associations between BMD and TMAO levels, we observed no correlation between them. However, the intervention period in the present study was relatively short.

SPARC [35] and decorin [36] levels may also affect skeletal muscle metabolism via different pathways. Increased expression of SPARC mediates some of the exercise-induced benefits both in terms of metabolic functions, including enhancement of glucose usage and oxidative phosphorylation, as well as tissue remodeling [35]. In addition, increased muscle decorin expression, correlates with improvement in leg press performance [36]. Taken together, SPARC and decorin could enhance the metabolic ability and structural (mechanical) properties of skeletal muscles. However, we found no associations between the changes in circulating SPARC and decorin levels and those in RM. This finding may indicate that exercise increases muscle strength by predominately locally derived mediators rather than circulating factors.

Notably, we observed a high correlation between the increase in SPARC and decorin levels with the increase in plasma TMAO levels. The associations of TMAO with inflammation, endothelial dysfunction, type 2 diabetes, central adiposity, hypertension, and cancer [37–39] have led to a growing interest in this metabolite. SPARC or decorin secretion is related to health benefits. Thus, modulation of SPARC production may have potential implications for postinfarction healing [40], and decorin has a protective role in several cardiac diseases, including atherosclerosis, hypertrophy, and myocardial infarction [41]. Moreover, SPARC and decorin might exert an anticancer effect by decreasing cancer cell proliferation, limiting migration and increasing apoptosis [42].

Numerous studies have examined the effect of training on BMD in postmenopausal women [43–46]. The results of the meta-analyses show that resistance training programs increase BMD when applied for ≥ 6 months [46], combined with high-impact or weight-bearing exercises [43], or in patients with osteoporosis and osteopenia [47]. In the present study, no clinically significant changes were found in the BMDs of the spine, hip, and total skeleton. Because the study protocol consisted of both exercise and supplementation, some favorable changes in the BMD could be expected. However, it was not possible to detect them in the short study period [46]. This finding is consistent with the lack of concurrent changes in the biochemical bone metabolism parameters in the study participants.

Limitations of the present study include the relatively short duration of the observation. Furthermore, no control group undergoing sham exercise was included. Finally, self-reported data were used to monitor dietary intake only once. Given the increase in the physical activity levels of the participants, their energy intake may have also increased, which could have resulted in changes in the dietary macronutrient composition.

## Conclusions

The administration of LC, as a dietary supplement during 12 weeks of RT program, increased the plasma TMAO levels and appeared to affect signaling molecules, as indicated by the increase in the resting SPARC and decorin levels. No significant modification in BMD and no differences between groups in RM could be due to the relatively short duration of the RT intervention. It is also not excluded that the same supplementation protocol without training program may induce different response in SPARC and decorin levels. Therefore, further studies are needed for a better definition of the effects of LC on SPARC, decorin, and TMAO levels in vivo in humans. Furthermore, it remains to be assessed whether LC supplementation, even that of short duration, may induce modifications, that can be preserved in the long term. Progress in understanding the mechanism of TMAO involvement in pathogenic processes may provide important information for antiaging strategies.

## Electronic supplementary material

Below is the link to the electronic supplementary material.

Supplementary Material 1

## Data Availability

The datasets used and analyzed during the current study are available from the corresponding author on reasonable request.

## List of Abbreviations

BMD: Bone mineral density
CRP: C-reactive protein
DKK1: Dickkopf-1
DXA: Dual-energy X-ray absorptiometry
FGF-23: Fibroblast growth factor-23
IGF-1: Insulin-like growth factor-1
IL-6: Interleukin-6
LC: L-carnitine
OPG: Osteoprotegerin
OPN: Osteopontin
RM: One-repetition maximum
RT: Resistance training
SOST: Sclerostin
SPARC: Secreted protein acidic and rich in cysteine/osteonectin
TC: Total carnitine
TMA: Trimethylamine
TMAO: Trimethylamine N-oxide
TNF-α: Tumor necrosis factor-α
