# Supplementary material for: Effect of a 3-month L-carnitine supplementation and resistance training program on circulating markers and bone mineral density in postmenopausal women: a randomized controlled trial
Source: Nutr Metab (Lond). 2023 Aug 2;20:32. doi: 10.1186/s12986-023-00752-1 (PMC10394783; doi:10.1186/s12986-023-00752-1)
Supplement: Supplementary file 1 — Supplementary Material 1 [file 12986_2023_752_MOESM1_ESM.docx]

**Supplementary material**

Figure S1 Scatter plots of correlations between the changes in serum decorin and SPARC levels (panel A), and changes in the decorin level correlated with total BMD (panel B).

r = 0.543

p = 0.003

A

r = 0.383

p = 0.049

B

Figure S2 Scatter plots of correlations between the changes in the levels of SPARC (panel A) or decorin (panel B) and TMAO levels

rho = 0.568

p = 0.002

A

rho = 0.574

p = 0.002

B

Table S1 Dietary composition of the participants. Data are presented as means ± SD

|  | PLA | LC | p-value |
| --- | --- | --- | --- |
| Energy (MJ/d) | 6.8 ± 1.0 | 6.8 ± 1.6 | 0.992 |
| CHO (g/d) | 225 ± 34 | 201 ± 42 | 0.139 |
| CHO (%) | 50 ± 6 | 46 ± 7 | 0.148 |
| Protein (g/d) | 74 ± 16 | 76 ± 16 | 0.785 |
| Protein (%) | 19 ± 3 | 19 ± 2 | 0.651 |
| Fat (g/d) | 57 ± 15 | 65 ± 28 | 0.402 |
| Fat (%) | 31 ± 5 | 35 ± 8 | 0.203 |
| SFA (g/d) | 20 ± 7 | 23 ± 11 | 0.373 |
| MUFA (g/d) | 21 ± 6 | 24 ± 11 | 0.350 |
| PUFA (g/d) | 11 ± 6 | 12 ± 6 | 0.648 |
| Minerals | | | |
| Na (mg/d) | 1745 ± 546 | 1715 ± 827 | 0.917 |
| K (mg/d) | 3421 ± 600 | 3272 ± 716 | 0.583 |
| Ca (mg/d) | 573 ± 192 | 565 ± 220 | 0.922 |
| P (mg/d) | 1315 ± 288 | 1302 ± 267 | 0.916 |
| Mg (mg/d) | 352 ± 87 | 329 ± 57 | 0.462 |
| Fe (mg/d) | 13.1 ± 2.4 | 12.8 ± 2.9 | 0.757 |
| Zn (mg/d) | 11.3 ± 2.4 | 11.8 ± 2.5 | 0.600 |
| Cu (mg/d) | 1.5 ± 0.3 | 1.3 ± 0.3 | 0.111 |
| Vitamins | | | |
| Beta-carotene (µg/d) | 4498 ± 2734 | 4330 ± 1689 | 0.860 |
| A (µg/d) | 1114 ± 407 | 1594 ± 1382 | 0.244 |
| D (µg/d) | 5.8 ± 5.3 | 4.4 ± 4.7 | 0.507 |
| E (mg/d) | 11 ± 4 | 10 ± 3 | 0.564 |
| B1 (mg/d) | 1.3 ± 0.3 | 1.1 ± 0.4 | 0.170 |
| B2 (mg/d) | 1.6 ± 0.2 | 1.7 ± 0.5 | 0.590 |
| Niacin (mg/d) | 19 ± 5 | 18 ± 4 | 0.547 |
| B6 (mg/d) | 2.0 ± 0.5 | 1.9 ± 0.5 | 0.793 |
| B12 (µg/d) | 5.0 ± 3.6 | 6.4 ± 5.0 | 0.442 |
| Folacin (µg/d) | 314 ± 83 | 310 ± 122 | 0.916 |
| Vitamin C (mg/d) | 126 ± 101 | 106 ± 44 | 0.542 |
